# Levodopa-induced dyskinesia in early-onset Parkinson’s disease (EOPD) associates with glucocerebrosidase mutation: A next-generation sequencing study in EOPD patients in Thailand

**DOI:** 10.1371/journal.pone.0293516

**Published:** 2023-10-31

**Authors:** Sekh Thanprasertsuk, Prasit Phowthongkum, Thitipong Hopetrungraung, Chalalai Poorirerngpoom, Tikumphorn Sathirapatya, Patsorn Wichit, Onanong Phokaewvarangkul, Kornkiat Vongpaisarnsin, Saknan Bongsebandhu-phubhakdi, Roongroj Bhidayasiri

**Affiliations:** 1 Department of Physiology, Faculty of Medicine, Chulalongkorn University, Bangkok, Thailand; 2 Cognitive Clinical & Computational Neuroscience (CCCN) Center of Excellence, Chulalongkorn University, Bangkok, Thailand; 3 Chula Neuroscience Center, King Chulalongkorn Memorial Hospital, Thai Red Cross Society, Bangkok, Thailand; 4 Division of Medical Genetics and Genomics, Department of Medicine, Faculty of Medicine, Chulalongkorn University, Bangkok, Thailand; 5 Excellence Center of Genomics and Precision Medicine, King Chulalongkorn Memorial Hospital, Thai Red Cross Society, Bangkok, Thailand; 6 Department of Medicine, Queen Savang Vadhana Memorial Hospital, Chonburi, Thailand; 7 Saraburi Hospital, Saraburi, Thailand; 8 Department of Forensic Medicine, Faculty of Medicine, Chulalongkorn University, Bangkok, Thailand; 9 Forensic Genetics Research Unit, Ratchadapiseksompotch Fund, Faculty of Medicine, Chulalongkorn University, Bangkok, Thailand; 10 Faculty of Physical Therapy, Huachiew Chalermprakiet University, Samut Prakan, Thailand; 11 Division of Neurology, Department of Medicine, Faculty of Medicine, Chulalongkorn University, Bangkok, Thailand; 12 Chulalongkorn Centre of Excellence for Parkinson’s Disease and Related Disorders, King Chulalongkorn Memorial Hospital, Bangkok, Thailand; 13 The Academy of Science, The Royal Society of Thailand, Bangkok, Thailand; University College London, UNITED KINGDOM

## Abstract

**Background:**

With the benefit of using next-generation sequencing (NGS), our aim was to examine the prevalence of known monogenic causes in early-onset Parkinson’s disease (EOPD) patients in Thailand. The association between clinical features, such as levodopa-induced dyskinesia (LID), and genotypes were also explored.

**Method:**

NGS studies were carried out for EOPD patients in the Tertiary-referral center for Parkinson’s disease and movement disorders. EOPD patients who had LID symptoms were enrolled in this study (n = 47). We defined EOPD as a patient with onset of PD at or below 50 years of age. LID was defined as hyperkinetic movements including chorea, ballism, dystonia, myoclonus, or any combination of these movements resulting from levodopa therapy, which could be peak-dose, off-period, or diphasic dyskinesias.

**Results:**

Pathogenic variants were identified in 17% (8/47) of the Thai EOPD patients, of which 10.6% (5/47) were heterozygous *GBA* variants (c.1448T>C in 3 patients and c.115+1G>A in 2 patients), 4.3% (2/47) homozygous *PINK1* variants (c.1474C>T) and 2.1% (1/47) a *PRKN* mutation (homozygous deletion of exon 7). The LID onset was earlier in patients with *GBA* mutations compared to those without (34.8±23.4 vs 106.2±59.5 months after starting levodopa, respectively, *p* = 0.001). LID onset within the first 30 months of the disease was also found to be independently associated with the *GBA* mutation (odds ratio [95% confidence interval] = 25.00 [2.12–295.06], *p* = 0.011).

**Conclusion:**

Our study highlights the high prevalence of *GBA* pathogenic variants in Thai patients with EOPD and the independent association of these variants with the earlier onset of LID. This emphasizes the importance of genetic testing in this population.

## Introduction

Parkinson’s disease (PD) is a neurodegenerative disorder pathologically characterized by the deposition of abnormal alpha-synuclein proteins in various areas of the brain, but primarily in the substantia nigra. In recent decades, several monogenic causes have been identified as associated with the pathogenesis of PD, such as pathogenic variants of alpha-synuclein (*SNCA*), leucine-rich repeat kinase 2 (*LRRK2*), parkin (*PRKN*), PTEN-induced putative kinase 1 (*PINK1*), and glucocerebrosidase (*GBA*) genes [1, 2]. Although a genetic cause appears to be uncommon among the overall population of PD, its prevalence is higher in patients with PD who have an earlier age at onset, that is, early-onset Parkinson’s disease (EOPD) [1–3]. Genetic studies of EOPD in Thailand, including studies of *LRRK2*, *GBA*, and TATA-binding protein genes, were performed traditionally by Sanger sequencing [4–6]. By taking advantage of next-generation sequencing (NGS) technology allowing rapid and high-throughput sequencing of DNA, fast and cost-effective genetic analysis can be performed, opening the door for a robust study of the genetic architecture of Thai EOPD patients.

In addition to earlier disease onset and a higher prevalence of genetic abnormalities, EOPD patients are suggested to have a slower disease progression, in terms of motor impairment, along with longer survival times from the disease onset, compared to late-onset PD patients [3, 7]. Levodopa sparing strategies; therefore, have been implemented in the pharmacological management of EOPD patients to delay the onset of motor complication arising from levodopa therapy, such as levodopa-induced dyskinesia (LID) [8, 9]. Delaying in levodopa therapy is considered a clinical equipoise, as studies have concluded that the development of LID is more closely related to the duration of the disease and the daily dose of levodopa, rather than the cumulative amount of levodopa received [10, 11]. Nevertheless, several studies have demonstrated that EOPD patients have a similar, or even earlier, onset of LID, compared to patients with late-onset PD [7, 12–14]. The underlying mechanisms of this earlier onset of LID, while PD motor symptoms progressed slowly, are not well established. Different genetic abnormalities are one of the possible factors being explored in this study.

We aimed to examine the prevalence of monogenic causes and the types of pathogenic variants in EOPD patients in Thailand using NGS. The clinical characteristics, including the onset of LID, of those patients identified as carrying genetic abnormalities were also explored to investigate any association with the genetic abnormalities.

## Materials and methods

### Participants

EOPD patients who are suffering from LID were enrolled from the outpatient clinic at Chulalongkorn Center of Excellence for Parkinson’s Disease and Related Disorders, King Chulalongkorn Memorial Hospital, Thailand (ChulaPD; www.chulapd.org) between June 2018 and July 2019. The diagnosis of PD was based on the Movement Disorder Society Clinical Diagnostic Criteria [15]. EOPD was defined as patients with PD whose age at onset of motor symptom ≤ 50 years [3, 5, 7]. We defined LID as various hyperkinetic movements including chorea, ballism, dystonia, myoclonus, or any combination of these movements that occur as a consequence of levodopa therapy, which could occur at a peak-dose, an off-period of levodopa effects, or diphasic dyskinesias in which hyperkinetic movements occur during both peak and trough levodopa effects [16, 17]. Demographic and clinical data including age, gender, age at the onset of motor symptom, family history of parkinsonism, disease duration, onset of LID from levodopa initiation, and presence of anosmia, constipation or REM sleep behavior disorder (RBD) were obtained from medical records along with patients’ confirmation. We classified LID onset within the first 30 months of levodopa initiation as an “early-onset LID”. Modified Hoehn and Yahr (HY) staging scale [18] and levodopa equivalent daily dose (LEDD) [19] were also assessed during the enrollment process.

Patients were provided verbal information related to the study including risks and benefits of genetic testing and were excluded if they, or their relatives, were uncomfortable with the test. Participants signed a written informed consent prior to finishing enrollment. The research protocol was approved by the Institutional Review Board of Faculty of Medicine, Chulalongkorn University, Thailand (approval number 023/61).

### DNA isolation

Peripheral blood samples were collected with Vacutainer^®^ EDTA tubes and immediately stored at -80°C until further processing. Two hundred microliters of the whole blood sample were subjected to DNA isolation with the QIAamp DNA blood mini kit (QIAGEN) using the manufacturer’s protocols and stored at -20°C. DNA concentration was determined using Qubit^™^ dsDNA HS Assay Kit with Qubit 2.0 Fluorometer (ThermoFisher Scientific).

### Library preparation and sequencing

Truseq^®^ Neurodegeneration Panel (Illumina Inc., San Diego, CA) was used to prepare the samples for sequencing according to the manufacture’s protocol. Sequencing was carried out on NextSeq 550 Instrument (Illumina Inc., San Diego, CA) with Nextseq mid output kit v2.5 (300 cycles).

### Data analysis

The FASTQ output was processed with a BWA aligner provided on *Base Space Sequencing Hub* (tools used are *BWA version 0*.*7*.*13* (https://github.com/lh3/bwa), *SAMtools version 1*.*3* (https://github.com/samtools/samtools), *Picard version 2*.*1*.*1* (https://github.com/broadinstitute/picard). The sequences were aligned against the human genome (hg19 version, Genome Reference Consortium GRCH37) reference sequence. Sequence variants, including single-nucleotide variations (SNVs), splice sites, and small indels in genes known to cause PD, were called using online Variant interpreter analysis software^®^ after upload to the webserver of the created vcf file. The genes list, using only genes with high evidence for Early onset and familial Parkinson’s disease (version 0.96 from PanelApp (Genomics England), includes 57 genes: *ATP13A2*, *ATP1A3*, *ATP1A3*, *ATP6AP2*, *ATP7A*, *C19orf12*, *C9orf72*, *CHCHD2*. *CLN3*, *CP*, *CSF1R*, *DCTN1*,*DNAJC12*, *DNAJC5*, *DNAJC6*, *FBX07*, *FMR1*, *FTL*, *GCH1*, *GRN*, *HD*, *LRRK2*, *LYST*, *MAPT*, *MECP2*, *OPA3*, *PANK2*, *DJ-1/PARK7*, *PDE8B*, *PDGFB*, *PDGFRB*, *PINK1*, *PLA2G6*, *POLG*, *PPP2R5D*, *PRKN*, *PRNP*, *PSEN1*, *PTRHD1*, *PTS*, *RAB39B*, *SLC20A2*, *SLC30A10*, *SLC39A14*,, *SLC6A3*, *SNCA*, *SPG11*, *SPR*, *SYNJ1*, *TH TUBB4A*, *TWNK*, *THAP1*, *TOR1A*, *TUBB4A*, *VPS13C*, *VPS35*, *WDR45*, *XPR1* and, in addition, *GBA*, which is listed in the Parkinson Disease and Complex Parkinsonism (version 1.68) by PanelApp. Genes where pathology was generally associated with structural variants or different mechanisms, such as nucleotide repeat expansions, were not analyzed and included *FMR1*, *HD*, *C9orf72*. Where we could not identify small nucleotide variants, we used the BAM file in Integrative Gene Viewers to identify only total exon or multi exons homozygous deletions and confirmed pathogenicity by alternative methods. A patient with homozygous exon deletion in *PRKN* was confirmed by an independent outside genetic laboratory (Invitae, California, USA). Variants were classified and interpreted using the standard variant classification of the American College of Medical Genetics and Genomics and the 2015 version of the American Molecular Pathology Society. The population databases used were the 1000 Human Genome Project database, the Genomes Aggregation database (GNOMAD), and the National Center for Biotechnology Information database of single-nucleotide polymorphisms (NCBI dbSNP). The mutation and variant databases used included Clinvar and the human genetic mutation database (HGMD). We reported genetic variants using the standard nomenclature recommended by the Human Genome Variation Society (http://www.hgvs.org/mutnomen). Regarding *GBA*, owing to technical complexities, the Gaucher Common Variants Tests is limited to 19 known pathogenic variants: c.84dupG (p.Leu29Alafs*18), c.115+1G>A (Splice donor), c.222_224delTAC (p.Thr75del), c.475C>T (p.Arg159Trp), c.595_596delCT (p.Leu199Aspfs*62), c.680A>G (p.Asn227Ser), c.721G>A (p.Gly241Arg), c.754T>A (p.Phe252Ile), c.1226A>G (p.Asn409Ser), c.1246G>A (p.Gly416Ser), c.1263_1317del (p.Leu422Profs*4), c.1297G>T (p.Val433Leu), c.1342G>C (p.Asp448His), c.1343A>T (p.Asp448Val), c.1448T>C (p.Leu483Pro), c.1504C>T (p.Arg502Cys), c.1505G>A (p.Arg502His), c.1603C>T (p.Arg535Cys), c.1604G>A (p.Arg535His); which are mappable and not lined in the NGS dead zone [20].

### Statistical analysis

Continuous and categorical data were presented as mean ± standard deviation (SD) and frequency (%), respectively. Comparisons between patients with and without pathogenic variants were done using unpaired t-test or Mann-Whitney U-test for continuous variables, and chi-square or Fisher’s exact test, where the expected numbers were small for categorical variables. Relevant clinical factors were assessed whether they were independently associated with, or were predictive of, the pathogenic variants using a logistic regression model. The exponentiated regression coefficient was estimated as an odds ratio (OR) and 95% confidence interval (CI) of each relevant clinical factor. Hosmer-Lemeshow goodness of fit test was applied in order to assess a goodness of fit of the logistic regression model. All statistical analyses were performed in SPSS statistics version 22 (IBM corporation, New York, USA). Statistical significance was considered when *p*-value < 0.05.

## Results

We enrolled 47 Thai EOPD patients in this study. Demographic data, which included the number and mean of all EOPD patients in the parameters of male, family history, age of the onset, disease duration and modified HY staging [15] were collected for statistical analysis. No statistical significance was found in the comparisons between patients without an identified pathogenic variant and patients with *GBA* pathogenic variants in the demographic data (Table 1). Data are missing in 1 patient due to the absence of specific details in their medical records. The first-presenting LID was peak-dose dyskinesia in all patients. We found a statistically significant difference when comparing the mean LID onset durations between patients without an identified pathogenic variant and patients with *GBA* pathogenic variants (Table 1). However, there were no statistical differences for LEDD, anosmia, constipation, and RBD when compared between patients without an identified pathogenic variant and patients with *GBA* pathogenic variants (Table 1).

**Table 1:** Demographic and characteristics of patients with and without identified pathogenic variants.

		Overall (n = 47)	Presence of pathogenic variants				p-value^b^
			None (n = 39)	*GBA* (n = 5)	*PINK1* (n = 2)	*PRKN* (n = 1)	
Male, n (%)		31 (66.0%)	26 (66.7%)	3 (60%)	2 (100%)	0	0.767
Positive family history, n (%)		10 (21.3%)	7 (17.9%)	1 (20%)	1 (50%)	1	0.911
Age at the onset, years		40.2±9.0	41.6±7.9	37.2±7.9	21.5±16.3	38.0	0.230
Disease duration, months		185.8±78.8	197.0±79.9	137.0±47.3	133.5±64.1	98.9	0.114
Modified HY staging, n (%)	1	2 (4.3%)	1 (2.6%)	0	0	1	0.961
	1.5	1 (2.1%)	1 (2.6%)	0	0	0	
	2	10 (21.3%)	8 (20.5%)	1 (20%)	1 (50%)	0	
	2.5	11 (23.4%)	9 (23.1%)	2 (40%)	0	0	
	3	11 (23.4%)	9 (23.1%)	1 (20%)	1 (50%)	0	
	4	5 (10.6%)	5 (12.8%)	0	0	0	
	5	7 (14.9%)	6 (15.4%)	1 (20%)	0	0	
LID onset, months		98.2±59.4^a^	106.2±59.5^a^	34.8±23.4	108.0±50.9	90.0	0.002*
LEDD, mg		1,099.1±651.6	1,127.1±666.2	1,129.0±683.9	885.0±77.8	287.5	0.914
Presence of anosmia, n (%)		16 (34.0%)	16 (41.0%)	0	0	0	0.139
Presence of constipation, n (%)		32 (68.1%)	29 (74.4%)	3 (60%)	0	0	0.432
Presence of RBD, n (%)		29 (61.7%)	25 (64.1%)	3 (60%)	1	0	0.798

^a^missing data (n = 1)

^b^statistical comparisons between patients without an identified pathogenic variant (n = 39) and patients with *GBA* pathogenic variants (n = 5)

*statistical significance (p < 0.05)

Pathogenic variants were identified in 8 (17.0%) of the 47 enrolled patients and consisted of 5 (10.6%) heterozygous *GBA* pathogenic variants, 2 (4.3%) homozygous *PINK1* pathogenic variants and 1 (2.1%) *PRKN* pathogenic variant (Table 2). Demographic data and clinical characteristics of patients with any pathogenic variants, and with each individual pathogenic variant are shown in the S1 Table and Table 1, respectively. Considering patients with *GBA* pathogenic variants (n = 5), compared to patients without an identified pathogenic variant (n = 39), the onset of LID was the only factor that was significantly different between these sub-populations (34.8±23.4 versus 106.2±59.5, respectively, *p* = 0.002). As there were only 2 patients with *PINK1* and 1 patient with *PRKN* pathogenic variants, we did not include these sub-populations as separate groups in the statistical comparison.

**Table 2:** Identified pathogenic variants.

Patient#	Sex	Gene	Variants	Inheritance pattern
**1**	F	*GBA*	c.115+1G>A	AD
**2**	M	*GBA*	c.115+1G>A	AD
**3**	F	*GBA*	c.1448T>C	AD
**4**	M	*GBA*	c.1448T>C	AD
**5**	M	*GBA*	c.1448T>C	AD
**6**	M	*PINK1*	Homozygous p.R492Ter	AR
**7**	F	*PINK1*	Homozygous p.R492Ter	AR
**8**	M	*PRKN*	Homozygous Exon 7 deletion	AR

We also performed logistic regression in order to determine whether the earlier onset of LID was independently associated with the presence of *GBA* pathogenic variants. Univariate and multivariate logistic regression models were then constructed (Table 3). Early-onset LID was found to be associated with the presence of *GBA* mutations (OR [95%CI] = 25.00 [2.12–295.06], *p* = 0.011), independent of gender, age at the onset of motor symptoms, modified HY stage, or daily LEDD. Again, patients with *PINK1* or *PRKN* pathogenic variants were not included in this statistical model.

**Table 3:** Logistic regression analyses of factors associated with the presence of *GBA* pathogenic variant.

Factors	Univariate analysis			Multivariate analysis		
	p-value	OR	95%CI	p-value	OR	95%CI
Sex (male)	0.768	0.75	0.11–5.06	0.774	0.70	0.06–8.24
Age at the onset	0.256	0.94	0.85–1.04	0.152	0.91	0.80–1.04
Modified HY staging	0.960	0.98	0.41–2.34	0.893	0.92	0.25–3.34
Early-onset LID^a^	0.009	17.50	2.05–149.15	0.011	25.00	2.12–295.06
LEDD	0.955	1.00	0.99–1.01	0.969	1.00	0.99–1.01

^a^Early-onset LID was defined as the LID that occurred within the first 30 months of the disease.

## Discussion

In our study, the 57 multigene panel using the NGS technique had a diagnostic yield of 17.0% to identify pathogenic variants in EOPD patients with LID. This is comparable to the probable prevalence of pathogenic variants in EOPD in Spain, which, when analyzed with a NGS panel of 17 genes, was reported to be about 22.2% [21]. No new pathogenic variants were identified. The prevalence of heterozygous *GBA* pathogenic variants (c.115+1G>A (x2) and c.1448T>C (x3)) associated with PD in this population was 10.6%, which is comparable to a previous study in Thailand [5]. This prevalence is also comparable to a study in Taiwan that analyzed *GBA* pathogenic variants in EOPD patients [22]. These emphasize that heterozygous *GBA* pathogenic variants increase the risk of early development of PD in Thai and probably East- Asian, populations. The risk of developing PD in a heterozygous carrier is about 10-fold that of the general population, or about 10%. Furthermore, in biallelic form of coexisting with other pathogenic variants, two of the common variants of *GBA* identified in these cohorts are known to cause Gaucher’s disease, a lysosomal storage disease with multisystemic involvement that includes the nervous, hematological and skeletal system.

**Fig 1:**
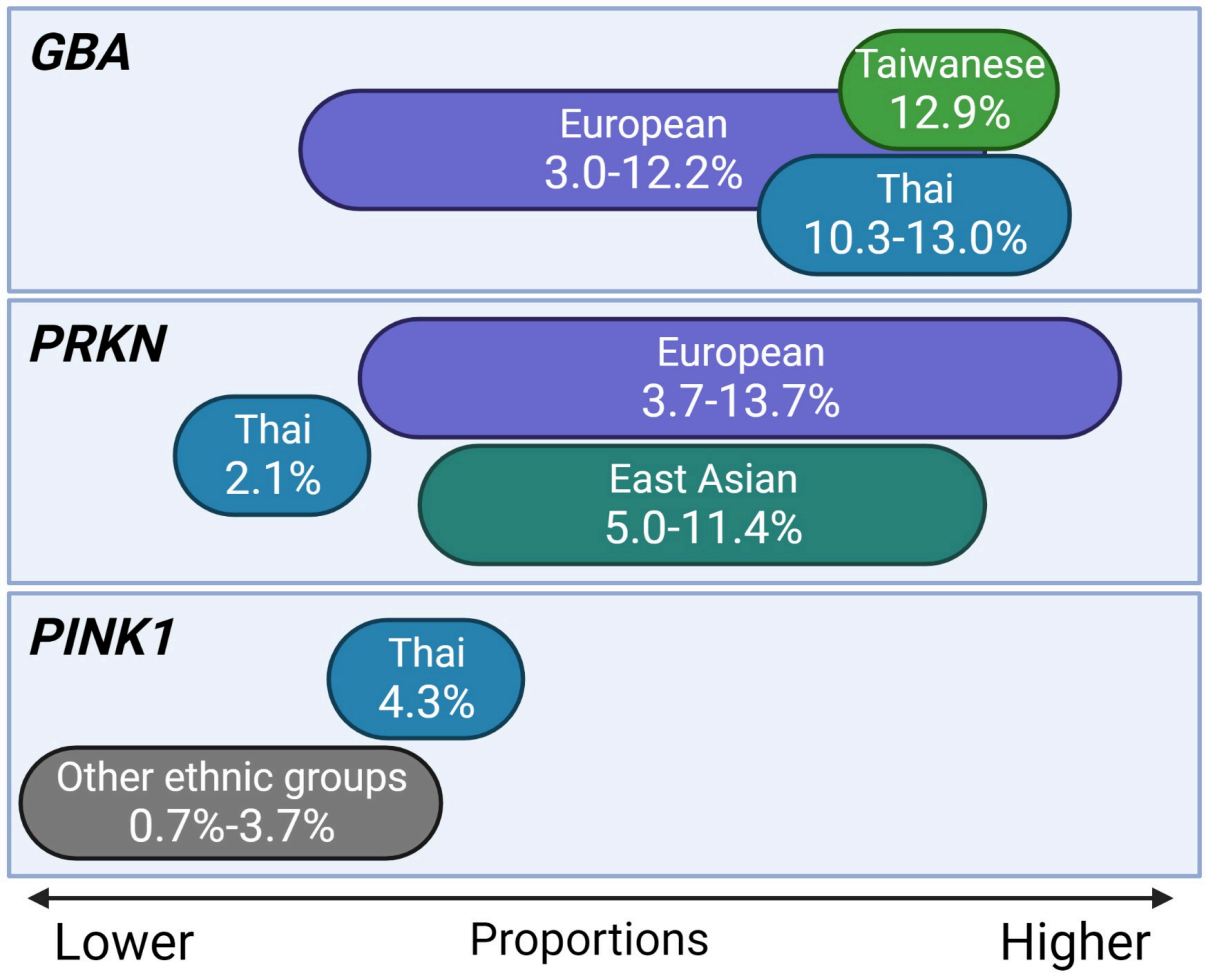
Simple illustration of *GBA*, *PRKN*, and *PINK1* pathogenic variants proportions in EOPD patients of Thai, East Asian, and European ethnic groups. *GBA* pathogenic variants are not uncommon among EOPD patients, especially in patients from Thailand and East Asia, such as Taiwanese, patients [5, 22, 27, 35]. *PRKN* pathogenic variants are quite common in EOPD patients of both European and East Asian ethnic groups [17, 19, 21, 23]. *PINK1* pathogenic variants are relatively rare in the general population of EOPD patients [23, 25].

However, in studies of European descents, *PRKN* is the most prevalent detected gene [21, 23, 24]. Also, the *LRRK2* variant, which is generally associated with familial late onset PD, was the second most prevalent in the Spanish cohort [21], but was absent from patients in our cohort. *PRKN* and *LRKK2* variants appear to be founder variants. The prevalence of the *PINK1* pathogenic variant (p.R492Ter) in this study was 4.3%, slightly higher compared to previous reports [23, 25, 26]. This same variant in an unrelated family is suggestive of a founder effect. However, this has yet to be confirmed with haplotype studies. It is also notable that the prevalence of *PINK1* pathogenic variants among EOPD patients is highly varied across studies, especially in the study of Asian population [23, 25, 26]. Simple illustration of *GBA*, *PRKN*, and *PINK1* pathogenic variants proportions in EOPD patients of Thai and other ethnic groups is shown in Fig 1.

Interestingly, we demonstrated that patients carrying *GBA* pathogenic variants had significantly earlier onset of LID compared to EOPD patients without an identified pathogenic variant. This finding is indirectly supported by a previous study reporting a higher frequency of LID and earlier development of levodopa-related motor complications among carriers of *GBA* pathogenic variants [27]. Based on our multivariate logistic regression model, the association is independent of the patient’s gender, age at the onset of PD, modified HY stage and LEDD. Age at enrollment was not included in the regression model, as this is a temporal parameter depending on the enrollment date rather than the natural history of the disease. For the same reason, disease duration was omitted from the model. In this case, parameters indicating the progression of the disease per unit time, that is, the disease progression rate, may be more appropriate to be entered in this analysis. Since the *GBA* mutation is not uncommon and is independently associated with the early development of LID, genetic testing in EOPD patients is crucial. Accordingly, extra-cautious pharmacological management should be applied in EOPD patients carrying *GBA* pathogenic variants, due to the potential for higher levodopa daily doses to result in LID [10].

The presynaptic and postsynaptic components of the nigrostriatal dopaminergic system, along with the non-dopaminergic neurotransmitter systems, were suggested to be involved in the pathophysiology of LID [16, 17, 28]. For the presynaptic component, a severe degree of striatal dopaminergic denervation causes a “short-duration response” to exogenous levodopa, resulting in pulsatile dopaminergic stimulations in the striatum and, consequently, LID [28]. However, this contrasts to patients with non-advanced PD, whose dopaminergic deficit is not severe and thus generally has a “long-duration response” to exogenous levodopa. For the post-synaptic component, continuous exposures of striatal neurons to pulsatile dopaminergic stimulations lead to changes in downstream signal transduction pathways, resulting in supersensitive dopamine receptors [28, 29]. Various clinical factors are reported to be associated with, or even predictive of, the development of LID. A factor that has been found to be a determinant of the onset of LID is the degree of nigrostriatal presynaptic dopaminergic deficit [30]. As the *GBA*-encoding lysosomal enzyme glucocerebrosidase decreases in patients carrying *GBA* pathogenic variants; its substrate, glucosylceramide, increases. Glucosylceramide could lead to an accumulation of α-synuclein, and α-synuclein accumulation could inversely cause a reduction in glucocerebrosidase enzymatic activity [31, 32]. Thus, in patients carrying *GBA* pathogenic variants, this vicious cycle possibly leads to a rapid progression of nigrostriatal dopaminergic deficit due to α-synuclein accumulation and may explain the rapid development of LID. Also, PD patients carrying *GBA* pathogenic variants generally have a more rapid motor progression [5, 33, 34], which can be accounted for the same explanation. It is noteworthy that patients with other α-synucleinopathy spectrum disorders, such as idiopathic RBD and dementia with Lewy bodies, are also reported to have a higher proportion of *GBA* pathogenic variants carriers compared to control subjects [31].

The benefits of identifying EOPD patients carrying *GBA* pathogenic variants in a clinical setting may not be limited to an individual level. Genetic counseling of a patient carrying a heterozygous *GBA* pathogenic variant is important, as biallelic *GBA* pathogenic variants in descendants cause Gaucher’s disease, a lysosomal storage disorder [32]. On the reverse side, counseling and genetic testing should be considered in parents whose children are diagnosed with Gaucher’s disease.

Some limitations of this study include the relatively small sample size collected from a single center in Thailand and the small number of patients with each genetic variant. Therefore, it is not possible to calculate meaningful statistics of each individual variant and the regression analyses results may not be perfectly precise. However, ChulaPD is a main tertiary referral center for PD in Thailand, where all study subjects were carefully diagnosed and managed by experienced neurologists of movement disorder.

Other limitations include the absence of whole-genome or whole-exome sequencing and analysis of copy number variations. For *GBA*, the absence of whole exon Sanger sequencing restricts the ability to validate the identified pathogenic variants by an alternative approach. This limitation also hinders the exploration of less common *GBA* variants that are exclusively detectable through this particular method.

Since *GBA* pathogenic variants are not uncommon [5, 21, 22], the findings from this single Thai cohort could still have clinical implications at a regional or probably global level. These implications include the importance of genetic testing in EOPD patients and the pharmacological management strategy in patients carrying *GBA* pathogenic variants. Further multi-center studies containing diverse populations would help to explore the association between the presence of *GBA* pathogenic variants and LID in patients with various demographic characteristics.

## Conclusions

In our study, the NGS technique had a diagnostic yield of 17.0% to identify pathogenic variants in EOPD with LID patients. These included the mutations of *GBA*, *PINK1* and *PRKN*, with the pathogenic variants of *GBA* appearing to be more common than the others. The earlier onset of LID was independently associated with the *GBA* mutation in this population. Thus, genetic testing is likely essential in EOPD patients. This is particularly important for those carrying *GBA* pathogenic variants who may benefit from extra-cautious pharmacological management, especially with regard to daily dose of levodopa.

## Supporting information

S1 Table Comparisons of demographic and clinical characteristics between patients with any pathogenic variants (n = 8) and without identified pathogenic variants (n = 39). (DOCX)
