# Supplementary material for: Levodopa-induced dyskinesia in early-onset Parkinson’s disease (EOPD) associates with glucocerebrosidase mutation: A next-generation sequencing study in EOPD patients in Thailand
Source: PLoS One. 2023 Oct 31;18(10):e0293516. doi: 10.1371/journal.pone.0293516 (PMC10617711; doi:10.1371/journal.pone.0293516)
Supplement: S1 Table — (DOCX) [file pone.0293516.s001.docx]

**Supplementary Table 1.** Comparisons of demographic and clinical characteristics between patients with any pathogenic variants (n=8) and without identified pathogenic variants (n=39)

|  | | **Presence of pathogenic variants** | | **p-value^b^** |
| --- | --- | --- | --- | --- |
|  |  | **No (n=39)** | ***Yes* (n=8)** |  |
| Male, n (%) | | 26 (66.7%) | 5 (62.5%) | 1.00 |
| Positive family history, n (%) | | 7 (17.9%) | 3 (37.5%) | 0.34 |
| Age at the onset, years | | 41.6±7.9 | 33.4±11.3 | 0.31 |
| Disease duration, months | | 197.0±79.9 | 131.4±45.2 | **0.028*** |
| Modified HY staging, n (%) | 1 | 1 (2.6%) | 1 (12.5%) | 0.822 |
|  | 1.5 | 1 (2.6%) | 0 |  |
|  | 2 | 8 (20.5%) | 2 (25.0%) |  |
|  | 2.5 | 9 (23.1%) | 2 (25.0%) |  |
|  | 3 | 9 (23.1%) | 2 (25.0%) |  |
|  | 4 | 5 (12.8%) | 0 |  |
|  | 5 | 6 (15.4%) | 1 (12.5%) |  |
| LID onset, months | | 106.2±59.5^a^ | 60.0±43.9 | **0.034*** |
| LEDD, mg | | 1,127.1±666.2 | 962.8±595.6 | 0.626 |
| Presence of anosmia, n (%) | | 16 (41.0%) | 0 | **0.038*** |
| Presence of constipation, n (%) | | 29 (74.4%) | 3 (37.5%) | 0.089 |
| Presence of RBD, n (%) | | 25 (64.1%) | 4 (50.0%) | 0.692 |

^a^missing data (n=1)

^b^statistical comparisons between patients without an identified pathogenic variant (n=39) and patients with *GBA* pathogenic variants (n=5)

*statistical significance (p < 0.05)
